# Cumulant expansion with localization: A new representation of the diffusion MRI signal

**DOI:** 10.3389/fnimg.2022.958680

**Published:** 2022-08-17

**Authors:** Maryam Afzali, Tomasz Pieciak, Derek K. Jones, Jürgen E. Schneider, Evren Özarslan

**Affiliations:** ^1^Leeds Institute of Cardiovascular and Metabolic Medicine, University of Leeds, Leeds, United Kingdom; ^2^Cardiff University Brain Research Imaging Centre (CUBRIC), School of Psychology, Cardiff University, Cardiff, United Kingdom; ^3^LPI, ETSI Telecomunicación, Universidad de Valladolid, Valladolid, Spain; ^4^Department of Biomedical Engineering, Linköping University, Linköping, Sweden; ^5^Center for Medical Image Science and Visualization, Linköping University, Linköping, Sweden

**Keywords:** diffusion MRI, cumulant expansion, localization regime, kurtosis, gradient strength

## Abstract

Diffusion MR is sensitive to the microstructural features of a sample. Fine-scale characteristics can be probed by employing strong diffusion gradients while the low *b*-value regime is determined by the cumulants of the distribution of particle displacements. A signal representation based on the cumulants, however, suffers from a finite convergence radius and cannot represent the ‘localization regime' characterized by a stretched exponential decay that emerges at large gradient strengths. Here, we propose a new representation for the diffusion MR signal. Our method provides not only a robust estimate of the first three cumulants but also a meaningful extrapolation of the entire signal decay.

## 1. Introduction

Diffusion magnetic resonance imaging (dMRI) is a non-invasive characterization technique whose sensitivity to tissue microstructure can be exploited to address numerous diagnostic and basic science challenges. Most commonly, this is accomplished by introducing a pair of diffusion sensitizing gradients (Stejskal and Tanner, 1965) into conventional measurements (such as spin echo), which makes it possible to probe the net displacements of water molecules undergoing random movements between the two gradient pulses. A quantity called the *b*-value (LeBihan and Breton, 1985) determines the level of diffusion sensitivity encoded in dMRI acquisitions. The *b*-value is given by *b* = *G*^2^δ^2^(Δ − δ/3), where δ and Δ are the duration and separation of the two pulses, respectively, and *G* = γ*g* with γ being the gyromagnetic ratio and *g* is the gradient strength.

Employing certain functional forms to represent the signal profiles, e.g., to approximate the signal's dependence on the *b*-value, has proven beneficial in dMRI. Importantly, such representations provide regularization, interpolation, and extrapolation of the signal, allowing for the analyses to be feasibly performed by utilizing limited amounts of data (De Luca et al., 2021). The particular functional form for the dMRI signal profile is determined based on the mathematical properties of the signal as predicted by the physics of diffusion and how it influences the dMRI signal.

One natural representation is achieved via the Maclaurin series expansion of the natural logarithm of the dMRI signal. Such an expansion is sometimes referred to as the cumulant expansion since the coefficients of different terms correspond to the cumulants of the net displacement distribution (Stepišnik, 1981; Liu et al., 2004; Jones, 2010; Kiselev, 2010). Measurements at small *b*-values reveal the apparent diffusion coefficient and tensor, which are voxel-averaged measures of the second moments of displacements and are probed in Diffusion Tensor Imaging (DTI) (Basser et al., 1994; Basser, 2002). DTI is the most widely used dMRI representation for characterizing anisotropic diffusion providing noninvasive markers of tissue state (Pierpaoli and Basser, 1996) and has been used for mapping anatomical connections between different regions of the brain (Conturo et al., 1999; Mori et al., 1999; Basser et al., 2000). This signal model is simple and successful as it is readily achievable with conventional clinical scanners.

As the *b*-value is increased, higher order moments of displacement become more prominent (Liu et al., 2004). The next term in the Maclaurin series contains the kurtosis of the net displacement distribution, leading to Diffusion Kurtosis Imaging (DKI) (Jensen et al., 2005). The diffusional kurtosis is a quantitative measure for the non-Gaussianity of the diffusion process (Jensen and Helpern, 2010). Since diffusional non-Gaussianity in brain tissue is strongly linked to microstructural tissue complexity, the kurtosis is of interest for investigating various neuropathologies (Steven et al., 2014; Marrale et al., 2016) as well as for studying both aging (Coutu et al., 2014; Billiet et al., 2015) and development (Paydar et al., 2014; Grinberg et al., 2017) in normal brain. The kurtosis can be characterized by several rotationally invariant metrics, such as the mean kurtosis (MK) and the kurtosis fractional anisotropy, which augment the more commonly-used DTI parameters of mean diffusivity (MD) and fractional anisotropy (Jensen and Helpern, 2010; Glenn et al., 2015b). In addition, the orientational dependence of the kurtosis in white matter can be exploited for fiber tractography (Lazar, 2010; Glenn et al., 2015a, 2016).

Retaining more terms in the Maclaurin series extends the validity of the representation toward larger *b*-values. In general, data at larger *b*-values offer not only a better description of the signal but also more parameters related to the tissue microstructure. Unsurprisingly, acquisitions at higher *b*-values have led to significant advances, e.g., in resolving more than one major fiber orientations within a voxel (Tournier et al., 2004; Tuch, 2004; Özarslan et al., 2006; Wedeen et al., 2012).

The cumulant expansion suffers from a serious deficiency; the Maclaurin series expansion has a limited radius of convergence leading to poor extrapolations of the signal at large *b*-values (*b* > 2, 000 *s*/*mm*^2^) (Jensen et al., 2005; Kiselev, 2010; Hutchinson et al., 2017) (The radius of convergence for cumulant expansion is the largest *b*-value for which the signal converges). To overcome this limitation, Özarslan et al. (2008) proposed an expansion in terms of a complete and orthogonal set of basis functions, and employed Hermite functions in particular. The three-dimensional adaptation of this approach has led to an alternative generalization of DTI, referred to as Mean Apparent Propagator MRI (MAP-MRI) (Özarslan et al., 2013). This approach provided superior ability to represent the dMRI signal (Ning et al., 2015; De Luca et al., 2021), and alternative measures of non-Gaussianity, anisotropy and zero-displacement probabilities. Recently, Saleem et al. (2021) validated the measures derived from high-resolution MAP-MRI via comparisons with histology data from the macaque brain. MAP-MRI was also successfully used in detecting changes induced by stroke (Boscolo Galazzo et al., 2018), Parkinson's disease (PD) (Le et al., 2020) and in distinguishing the grades of gliomas (Wang et al., 2021; Sun et al., 2022), comparing the microstructural integrity of the corticospinal tract (CST) between glioma patients with and without motor epilepsy (Wang et al., 2022) and detecting amyotrophic lateral sclerosis (ALS)-related white matter (WM) alterations (Chen et al., 2021).

When strong and long gradient pulses are applied, the signal decay, *E*(*b*), is described by a stretched exponential function *E*(*b*) ∝ *e*^−^(*bD*)^1/3^, which is the characteristic feature of the *localization regime* (Stoller et al., 1991). In localization regime, diffusion in a strong constant gradient suppresses the signal everywhere except next to pore walls. More specifically, this regime is realized when the dephasing length (the typical length scale over which a spin must travel to dephase by 2π radians) $\ell_{g}={\left(D_{0}/G\right)}^{1/3}$ is shorter than the geometric length scales within the specimen as well as the diffusion distance $\ell_{\delta }=\sqrt{D_{0}\delta }$, where *D*_0_ is the bulk diffusivity of the fluid. In this regime, the transverse magnetization far from the boundaries of the medium vanishes and only a thin layer near the boundaries contributes to the measured signal (Moutal et al., 2019). Hürlimann et al. (1995) and Moutal et al. (2019) both reported the experimental observation of the localization regime. In parallel with the availability of stronger gradient strengths, studies investigating the localization regime have intensified in recent years leading to a number of interesting predictions (de Swiet and Sen, 1994; Grebenkov, 2014, 2018; Grebenkov et al., 2017; Herberthson et al., 2017; Ning et al., 2018). For example, the localization of the magnetization near the boundaries was shown to enhance the sensitivity of the signal to differences in membrane permeability (Grebenkov, 2014; Grebenkov et al., 2017). Williamson et al. (2019) and Cai et al. (2022) developed magnetic resonance (MR) methods for measuring the tissue microstructure and membrane permeability of live and fixed excised neonatal mouse spinal cords on a one-sided NMR scanner under an extremely large magnetic field gradient. Lee et al. (2021) detected the localization regime *in vivo* using a Connectom scanner and estimated the soma size in cortical brain gray matter.

We note that although MAP-MRI does not suffer from the convergence issue encountered in DKI, having finite number of terms in the MAP-MRI series representation makes the localization regime inaccessible since the basis functions have an exponentially decaying tail. When the reconstruction of the average propagator is concerned, this leads to a smoothing of the actual propagator. In this work, we provide an extension of cumulant expansion that can represent the diffusion-weighted signal in the localization regime as well. Our report is organized as follows. First, we present in Section 2 the signal representation that we propose. Section 3 depicts the results obtained from previously-published as well as new data. The characteristic stretched-exponential decay of the signal in the localization regime is observed at moderately high gradients and in various geometries, including unbounded diffusion outside obstacles. We discuss the implications of these results and conclude the paper in Section 4.

## 2. Methods

### 2.1. Theory

We consider acquisitions featuring diffusion gradients applied either in the same direction (single-direction acquisition), or in different directions uniformly-distributed on the surface of the sphere, where orientational-averaging is to be employed over each *b*-value shell. In either case, one obtains a one-dimensional signal profile, *E*(*q*). To represent the signal attenuation, we propose the following expression


(1) $$E(q)=\text{exp}(-{\left(qr\right)}^{2/3}({\left(qu\right)}^{\frac{4}{3}e^{-q^{6}p^{6}}}-{\left(qv\right)}^{\frac{10}{3}e^{-q^{6}p^{6}}}+{\left(qw\right)}^{\frac{16}{3}e^{-q^{6}p^{6}}})),$$

where *r*, *u*, *v*, *w*, and *p* are the parameters of the representation. The asymptotic (large-*q*) behavior of this function is governed by the desired stretched exponential decay since


(2) $$\begin{array}{l} \underset{q\to \infty }{\lim}\frac{E\left(q\right)}{e^{-{\left(qr\right)}^{2/3}}}=1 \end{array}$$

We note that the asymptotic behavior (of an expression) becomes increasingly accurate as a variable approaches a limit, usually infinity.

At small *q*-values, the function is given by


(3) $$\text{ln }E(q)=-q^{2}r^{2/3}u^{4/3}+q^{4}r^{2/3}v^{10/3}-q^{6}r^{2/3}w^{16/3}+O\left(q^{8}\right).$$

where the argument of O(·) denotes the growth rate of the remaining term.

This small-*q* behavior is consistent with DKI while the representation exhibits the stretched exponential decay at large-*q*, characteristic of the localization regime. Thus, our signal representation works at both low and high *b*-values. The parameters *r*, *u*, *v*, *w*, and *p* provide this flexibility for our representation. In the given signal representation, *r* determines the asymptotic behavior. For a given *r*, *u* determines the 2^nd^ cumulant, *v* determines the 4^th^ cumulant, and *w* is related to higher order cumulants.

In this study, the representation in Equation (1) is fitted to dMRI signals for two scenarios, namely, to orientationally-averaged signal (Afzali et al., 2021) and to single-direction acquisitions. The parameters *r, u, v, w*, and *p* were obtained by solving the nonlinear optimization using the trust-region algorithm implemented in MATLAB (The MathWorks, Inc., Natick, MA).

### 2.2. Relationship between DKI and our proposed method

The form of the signal for diffusion kurtosis imaging (DKI) is given by:


(4) $$\begin{array}{l} \text{ln }E\left(q\right)=-q^{2}\left(\Delta -\delta /3\right)D+\frac{1}{6}q^{4}{\left(\Delta -\delta /3\right)}^{2}D^{2}K, \end{array}$$

where *D* is the apparent diffusion coefficient and *K* is the mean kurtosis. Comparing Equations (3) and (4), we have:


(5) $$\begin{array}{l} D=\frac{r^{2/3}u^{4/3}}{\left(\Delta -\delta /3\right)}, \end{array}$$

and


(6) $$\begin{array}{l} K=\frac{6r^{2/3}v^{10/3}}{{\left(\Delta -\delta /3\right)}^{2}D^{2}}. \end{array}$$

### 2.3. Experimental data

The experimental data representing gas diffusion with hyperpolarized xenon-129 continuously flowing through different geometries of three physical phantoms were used to validate the proposal: (1) parallel plates separated by a distance of 3 mm (Figure 4 from Moutal et al., 2019), (2) cylindrical tubes with a cylinder of a diameter 3.8 and 2 mm (Figures 6, 7 from Moutal et al., 2019), and (3) cylindrical rods on a square grid with a cylinder of a diameter 3.8 mm (Figures 10 (a) and 10 (b) from Moutal et al., 2019).

### 2.4. *In vivo* data

- Two healthy participants were scanned using a 3T Connectom MR imaging system with a maximum gradient strength of 300 mT/m (Siemens Healthineers, Erlangen, Germany). Two acquisition protocols were used: (1) the first protocol comprised six *b* = 0 and 11 non-zero shells at (*b* = 0.4, 0.8, 1.2, 2, 3, 4, 6, 8, 10, 12, 15ms/μm^2^) along (16, 16, 21, 31, 21, 21, 31, 31, 31, 31, 46) gradient directions, respectively (Knutsson, 2018; Afzali et al., 2021), and (2) the second protocol included the data acquired in a single direction, ${\left(1/\sqrt{3},1/\sqrt{3},1/\sqrt{3}\right)}^{⊺}$, following the same *b*-value distribution as the former one. Contrary to the former protocol, in the latter case, the measurements were repeated with the same number of directions per *b*-value as in the first protocol. In total, 66 axial slices were acquired with 2 mm isotropic voxel size, matrix size of 106 × 106, TE/TR = 55/4,000 ms, Δ/δ = 23/12 ms and partial Fourier factor of 6/8. The phase variation in each complex-valued diffusion weighted image was removed using the method proposed by Eichner et al. (2015). Real-valued diffusion-weighted images were then corrected for Rician bias (Koay et al., 2012), Gibbs ringing artifacts (Kellner et al., 2016), eddy currents and subject motion using FSL EDDY (Andersson and Sotiropoulos, 2016). The orientationally-averaged signal was normalized based on the *b* = 0 in each voxel.
- Additionally, the data points from Figure 3 in Lee et al. (2021) representing the cortical gray matter were used for experimental verification of the proposal. The data come from the scanning of two healthy subjects using a Connectom scanner (Siemens Healthineers) with a gradient strength at 50-275 mT/m under two acquisition setups: (1) two *b* = 0 and 10 non-zero shells at (*b* = 0.4, 0.8, 1.2, 2, 3, 4, 6, 8, 10, 12ms/μm^2^), Δ/δ = 22/11 ms, and (2) two *b* = 0 and 10 non-zero shells at (*b* = 0.6, 1.2, 1.8, 3, 4.5, 6, 9, 12, 15, 18ms/μm^2^), Δ/δ = 24/13 ms. In both cases, the data was acquired along 32 gradient directions per shell with a 2 mm isotropic voxel size, TE/TR = 62/5,200 ms and partial Fourier factor of 6/8.

## 3. Results

**Figure 1:**
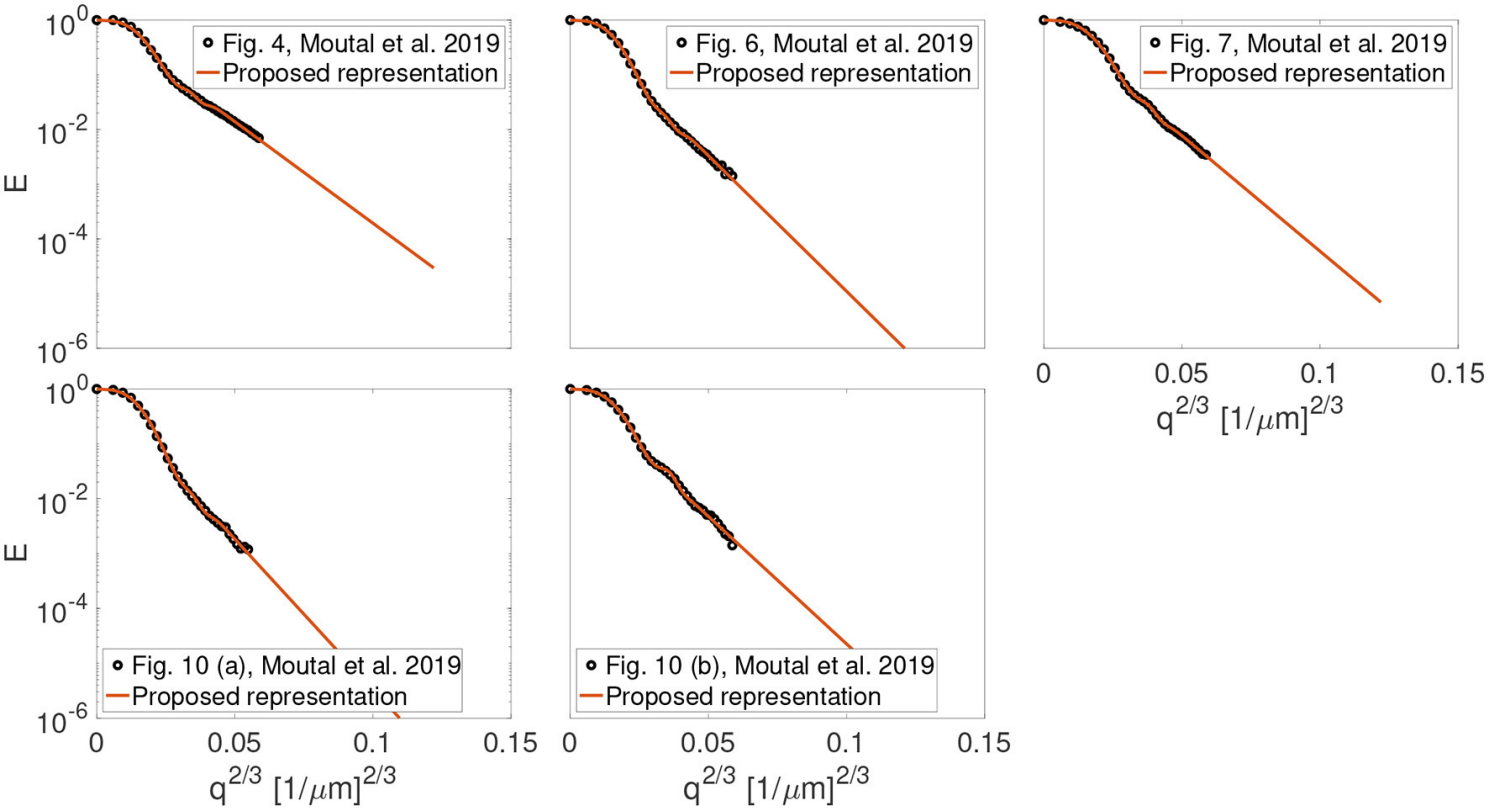
The result of fitting the proposed representation to the experimental data from Figures 4, 6, 7, 10 (a) and, 10 (b) in the work by Moutal et al. (2019).

Figure 1 shows the result of fitting the proposed representation given by Equation (1) to the experimental data from Figures 4, 6, 7, 10 (a) and 10 (b) in the work by Moutal et al. (2019). The extrapolated signal follows the trend of localization regime (−ln(*E*) ∝ *q*^2/3^).

**Figure 2:**
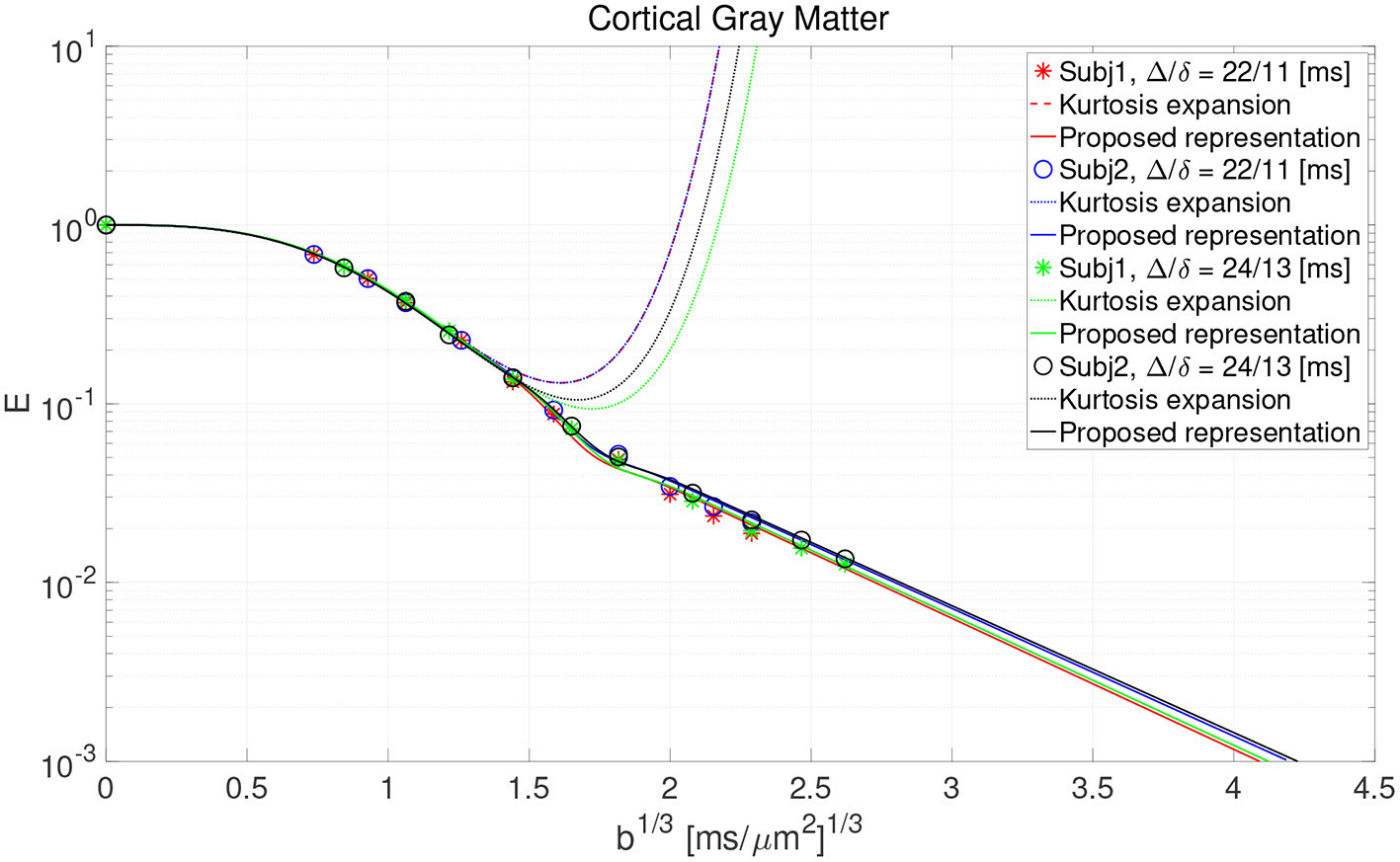
The result of fitting the proposed representation to the data from cortical gray matter voxels in the work by Lee et al. (2021).

The result of fitting the proposed representation to data from cortical gray matter in Lee et al. (2021) is illustrated in Figure 2. As shown, the representation based on the cumulant expansion (diffusion kurtosis imaging, DKI; Jensen et al., 2005) diverges at moderate *q*-values, while our representation fixes this problem.

**Figure 3:**
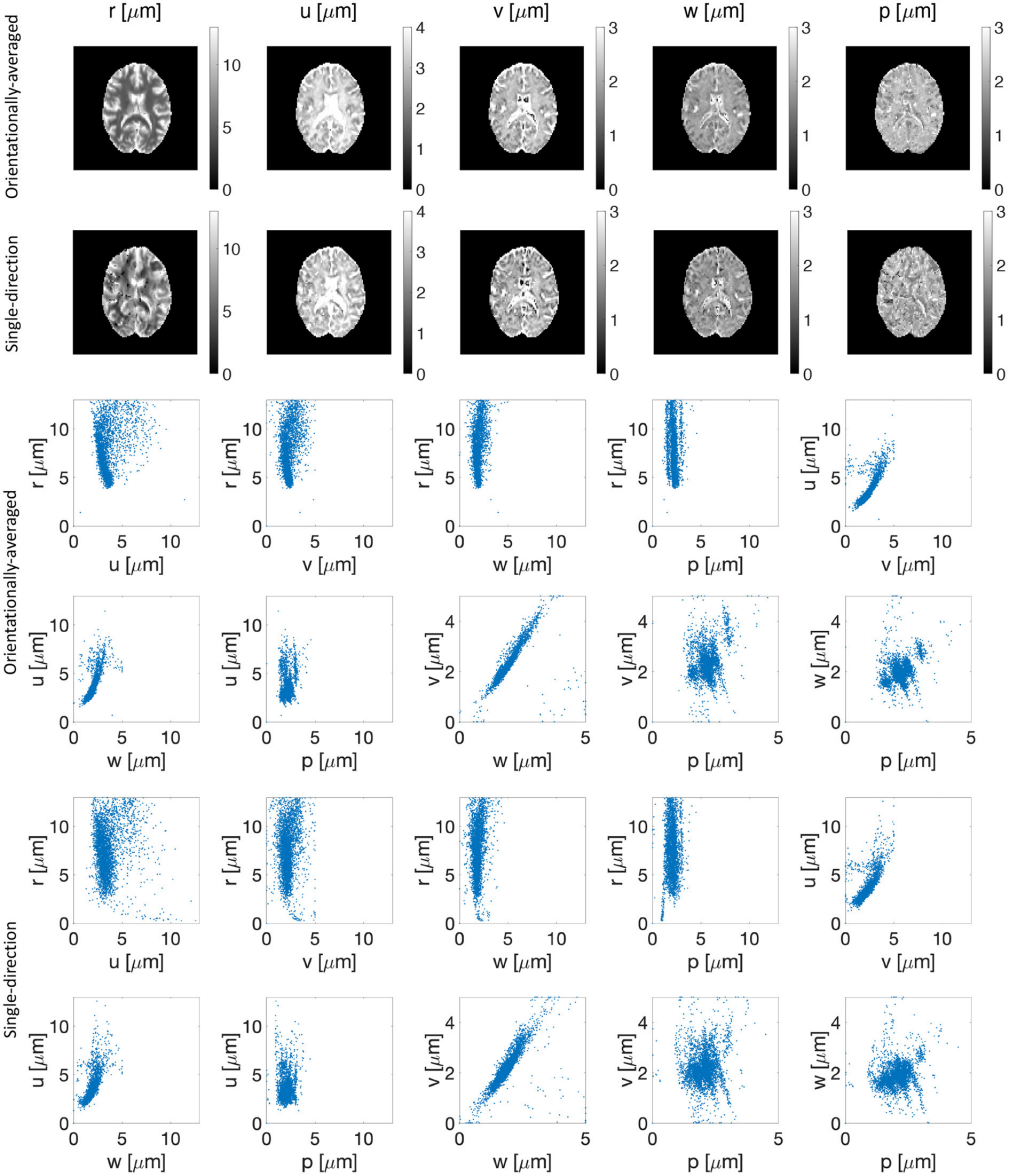
Estimated parameters (*r*, *u*, *v*, *w*, and *p*) of the representation for the orientationally-averaged (first row) as well as the single-direction signal (second row). The third to sixth rows show the scatter plots of proposed representation parameters vs. each other for the orientationally-averaged (rows three and four) and single-direction signal (rows five and six).

Figure 3 shows the estimated parameters (*r*, *u*, *v*, *w*, and *p*) of the representation for both orientationally-averaged signal and single-direction signals. No significant correlation between the estimated parameter *r* and the other parameters of the representation is seen in the scatter plots in Figure 3. Note further that the estimated *r* map from the brain image is also smooth.

**Figure 4:**
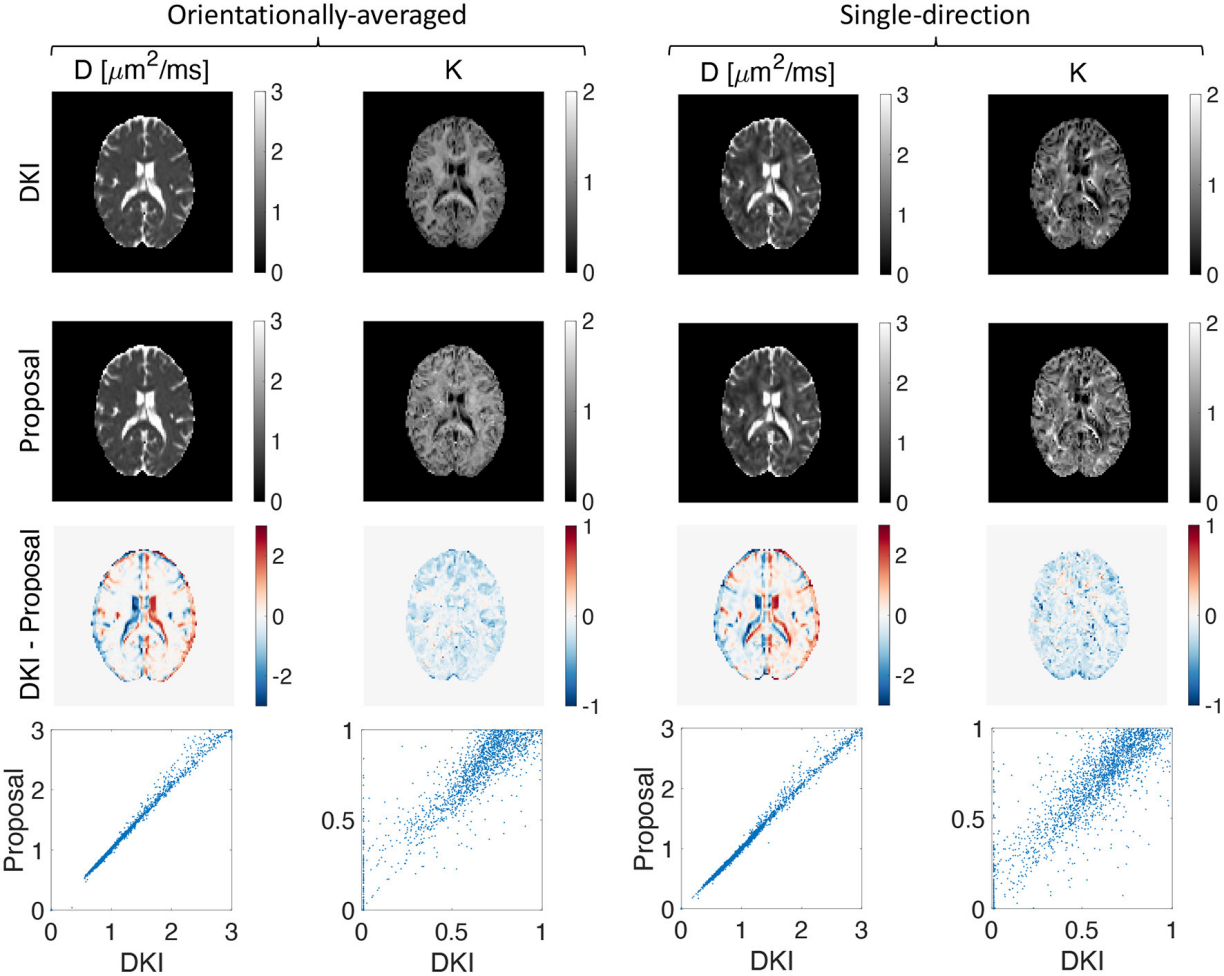
Estimated apparent diffusion coefficient and kurtosis using DKI (first row) and our proposed representation (second row), the difference between the estimated *D* and *K* using DKI and our proposed method (third row), and the scatter plot of the proposed method vs. DKI for *D* and *K* (fourth row) for both orientationally-averaged and single-direction scenarios.

We extracted the apparent diffusion coefficient and kurtosis using DKI and the low-*q* approximation of our proposed representation. A good agreement between the maps from DKI and our proposed method is observed as illustrated in Figure 4.

## 4. Discussion and conclusion

Diffusion MRI has become a key tool in diagnostic medicine as well as material science (Callaghan, 1993; Tuch et al., 2003; Frahm et al., 2004; Grebenkov, 2007; Price, 2009; Le Bihan and Johansen-Berg, 2012). However, most of the works are based on the Gaussian phase approximation (weak gradient amplitudes, i.e., low *b*-values) while only a few studies investigated the localization regime theoretically (Stoller et al., 1991; de Swiet and Sen, 1994) and experimentally so far (Hürlimann et al., 1995; Moutal et al., 2019; Williamson et al., 2019; Lee et al., 2021).

In the localization regime, the dMRI signal is attenuated by one or more orders of magnitude and therefore there is a need for an experimental setup with high signal-to-noise ratio. Substantial advances in MRI scanner technology compared to the first experiments related to the localization regime by Hürlimann et al. (1995) making it experimentally tractable. The limitation, however, is that the Gaussian is intermingled with stretched-exponential decay of diffusion weighted signal. By applying strong gradients, one can separate free water and restricted water as free water decays exponentially with *b*^1^ while the restricted water decays exponentially with *b*^1/3^ (Grebenkov, 2018).

The Gaussian phase approximation may be invalidated by the current trend in increasing *b*-value above values of *b* > 2, 000*s*/*mm*^2^ and therefore there is a need for appropriate mathematical tools and better interpretation of signal attenuation mechanism in the presence of strong gradients.

We have proposed a representation consistent with the localization regime which we tested on the experimental data in three geometries: slab, cylinder, and array of circular obstacles (rods) (Moutal et al., 2019) as well as *in vivo* brain data. Slab and cylinder are examples of confined geometries and can be used as a model for intracellular space. In most of the previous studies, a Gaussian model was assumed for the extracellular space and multiple pools were used to explain the non-Gaussian effects. One may assume that the *b*-values are small enough that the Gaussian phase approximation is valid or the obstacles can be considered as a medium with an effective diffusivity *D* such that the diffusion process is Gaussian. The latter case is only applicable in the very long time limit, as it has been discussed by Novikov et al. (2014).

Our representation of the diffusion MR signal reproduces DKI at low gradient strengths while capturing the stretched exponential behavior consistent with the localization regime at large gradient strengths. We have thus achieved a meaningful extrapolation of the signal, overcoming a serious deficiency of the cumulant expansion.

Note that fitting a stretched exponential function to the entire diffusion-weighted signal profile is problematic as its low-*q* behavior suggests diverging values of quantities such as the apparent diffusion coefficient and kurtosis as pointed out by Özarslan et al. (2012). Our representation has the stretched exponential tail, yet guarantees finite values for these quantities.

To take advantage of the localization regime, for example for estimating the soma size as done by Lee et al. (2021), it is difficult to decide on an appropriate range of *b*-values for fitting the asymptotic form. Having a good representation would alleviate this problem.

As demonstrated by Dela Haije et al. (2020), DKI estimation could benefit significantly from constrained estimation schemes enforcing convexity conditions that arise from the underlying stochastic process. We intend to employ such methods in future work.

In conclusion, we introduced a new diffusion MRI signal representation that could perform well in the entire range of gradient strengths, and thus overcomes the well-known challenges associated with the commonly-employed representations. Due to its ability to accommodate the relevant mathematical features that the signal profiles are expected to have, the proposed representation could improve our ability to reliably probe the information available in the signal using fewer measurements. It can achieve the same in studies involving large gradient strengths to determine the fine structural characteristics of the tissue which are available in the localization regime.

## Data availability statement

The raw data supporting the conclusions of this article are available at this link: http://doi.org/10.17035/d.2022.0215863820.

## Ethics statement

The studies involving human participants were reviewed and approved by the School of Psychology Research Ethics Committee, Cardiff University. The patients/participants provided their written informed consent to participate in this study.

## Author contributions

MA: methodology, software, formal analysis, investigation, data curation, writing—original draft, and visualization. TP: formal analysis, investigation, and writing—review and editing. DJ: resources, writing—review and editing, supervision, project administration, and funding acquisition. JS: resources, writing—review and editing, supervision, project administration, and funding acquisition. EÖ: conceptualization, methodology, validation, formal analysis, investigation, writing—original draft, writing—review and editing, and project administration. All authors contributed to the article and approved the submitted version.

## Funding

This research was funded in whole, or in part, by a Wellcome Trust Investigator Award (219536/Z/19/Z, 096646/Z/11/Z) and a Wellcome Trust Strategic Award (104943/Z/14/Z), the Swedish Foundation for Strategic Research (RMX18-0056), Linköping University Center for Industrial Information Technology (CENIIT), Sweden's Innovation Agency (VINNOVA) ASSIST, and Analytic Imaging Diagnostic Arena (AIDA). This work was also supported by the British Heart Foundation, UK (SI/14/1/30718), EPSRC (EP/M029778/1), and The Wolfson Foundation. TP acknowledges the Polish National Agency for Academic Exchange for the grant PPN/BEK/2019/1/00421 under the Bekker programme and the Ministry of Science and Higher Education (Poland) under the scholarship for outstanding young scientists (692/STYP/13/2018).

## Conflict of interest

EÖ is a shareholder of Spin Nord AB. The remaining authors declare that the research was conducted in the absence of any commercial or financial relationships that could be construed as a potential conflict of interest.

## Publisher's note

All claims expressed in this article are solely those of the authors and do not necessarily represent those of their affiliated organizations, or those of the publisher, the editors and the reviewers. Any product that may be evaluated in this article, or claim that may be made by its manufacturer, is not guaranteed or endorsed by the publisher.
